# Combined Effects of Dietary Guidance and Prosthodontic Rehabilitation on Nutritional Biomarkers in Edentulous Patients: A Randomized Controlled Trial

**DOI:** 10.7759/cureus.76215

**Published:** 2024-12-22

**Authors:** Haruka Sako, Yuka Abe, Yuriko Kusumoto, Ranko Kawata, Takashi Matsumoto, Takumi Yokoi, Maoko Hara, Junichi Furuya, Kazuyoshi Baba

**Affiliations:** 1 Department of Prosthodontics, Graduate School of Dentistry, Showa University, Tokyo, JPN; 2 Division of Prosthodontics, Department of Prosthodontics, School of Dentistry, Showa University, Tokyo, JPN; 3 Department of Oral Function Management, Graduate School of Dentistry, Showa University, Tokyo, JPN

**Keywords:** amino acid, complete dentures, dietary counseling, masticatory function, nutritional advice, nutritional status, removable dentures

## Abstract

Background

Previous studies have suggested that providing dietary guidance along with denture treatment may improve dietary diversity in edentulous patients; however, none have examined the effects on nutritional blood biomarkers. This study investigated the effects of individualized dietary guidance combined with complete denture treatment on nutritional blood biomarker levels, dietary intake, and masticatory function.

Materials and methods

This was a prospective, randomized, open-label, controlled trial. Patients seeking new dentures with at least one edentulous arch were randomly allocated to Group A (denture treatment only) or Group B (dietary guidance followed by denture treatment). Individualized dietary guidance was provided exclusively to Group B by dentists based on baseline assessments. Baseline and post-treatment assessments included blood tests, a dietary survey using the Brief-type Self-Administered Diet History Questionnaire (BDHQ), occlusal force measurements using pressure-sensitive film, and masticatory performance tests using gummy jelly. The results from the baseline and post-treatment assessments were compared within each group, and exploratory comparisons of changes were conducted between groups.

Results

A total of 39 individuals completed the protocol (20 in Group A and 19 in Group B; mean age 73.4 ± 10.6 years; 17 males, 22 females). Both groups showed a significant increase in occlusal force after treatment (*P* = 0.007 for Group A; *P* = 0.011 for Group B), while no improvement in blood biomarkers was observed. In Group B, the intake of fish with bones (*P* = 0.035) and non-oily fish (*P* = 0.030), as evaluated using the BDHQ, significantly increased after treatment. Group B also showed significantly greater increases than Group A in methionine (*P* = 0.012), lysine (*P* = 0.048), and total essential amino acids (*P* = 0.029), as evaluated by blood tests, as well as the intake of fish with bones (*P* = 0.043).

Conclusion

Although denture treatment may increase occlusal force, combining dietary guidance with prosthetic treatment appears to further enhance dietary habits and support the nutritional status of edentulous patients.

## Introduction

Tooth loss and impaired masticatory function can increase the risk of malnutrition [1,2], which may contribute to frailty, osteoporosis, and muscle weakness [3] and has been linked to sarcopenia [4]. In terms of dietary habits, individuals with fewer teeth tend to consume fewer vegetables and less dietary fiber while increasing their intake of cholesterol, saturated fat, and calories [5], along with sweet and sugary snacks [6]. As more teeth are lost, preferences tend to shift toward softer, high-fat, and high-calorie foods [7], which may lead to reduced dietary diversity and a higher risk of physical frailty [8]. Conversely, prosthetic treatment for missing teeth improves masticatory function, oral health-related quality of life [9], and food acceptability [10], which may improve nutritional status.

However, prosthetic treatment alone may not significantly affect patients' nutritional status because previous studies did not find significant improvements in blood test results (e.g., serum albumin, prealbumin, and zinc levels), Mini Nutritional Assessment (MNA) scores, a tool designed to assess the nutritional status of older adults, among nursing home residents [11], or nutrient intake among outpatients [12]. It has been suggested that combining dietary guidance with prosthetic treatment may positively influence dietary habits [13,14]. Specifically, providing individualized dietary guidance from dietitians [13] or dietary guidance via uniform pamphlets [14,15], in addition to complete denture treatment, has been reported to improve the intake of certain foods and MNA scores. This combined approach has also shown positive effects in patients with removable partial dentures [16,17]. These reports suggest that combining prosthetic treatment with dietary guidance may improve nutritional status. However, most studies have primarily assessed dietary intake through questionnaires or dietary records and have not evaluated blood biomarkers.

The Global Leadership Initiative on Malnutrition (GLIM) criteria, which include malnutrition screening and assessment, are currently recommended for diagnosing malnutrition in adults [18]; however, this process requires multiple steps and is time-intensive and complex. Questionnaire-based nutritional screening tools, such as the MNA short form, are noninvasive and easy to use; however, the application of the MNA short form is limited to individuals aged 65 years or older who are capable of self-reporting. In contrast, blood biomarkers provide a rapid and objective assessment of the nutritional and systemic status in clinical settings, making them effective for continuous nutritional monitoring and management across all age groups. Key biomarkers of protein status, such as serum albumin, prealbumin, and total serum protein, are commonly used [19], although they can be influenced by inflammation and require the concurrent measurement of inflammatory markers such as C-reactive protein (CRP) for accurate interpretation. The hemoglobin level is also often assessed, especially in older adults prone to anemia [19]. Moreover, cholesterol levels, which reflect diet and metabolic health, and lymphocyte counts, which are indicators of immune function, also aid in nutritional assessments [20]. Additionally, essential amino acid (EAA) levels provide insights into protein quality, as EAAs must be obtained from the diet [21].

Dietary surveys, such as the Brief-type Self-administered Diet History Questionnaire (BDHQ), are valuable for assessing dietary diversity [14]. To assess whether nutritional status improves following dietary guidance or prosthetic treatment due to changes in ingested foods, blood biomarkers and dietary intake should be examined. This includes measuring specific blood biomarkers that reflect nutrient absorption and utilization, as well as tracking the types and quantities of foods consumed to evaluate their potential impact on overall nutrition. However, no studies have explored the effects of combining dietary guidance with prosthetic treatment on these nutritional blood biomarkers in edentulous patients.

Therefore, this study aimed to explore changes in blood biomarkers that may reflect the nutritional status and dietary and nutrient intake by comparing two groups of outpatients seeking complete denture treatment: one group received new dentures only and the other received individualized, simple dietary guidance and new complete dentures. The null hypothesis was that there would be no differences in the changes in blood biomarkers, dietary intake, or masticatory function between the groups.

## Materials and methods

Participants

This was a prospective, randomized, open-label, controlled trial. Patients who visited a university hospital in Tokyo, Japan, between July 2023 and September 2024 were included if they were edentulous in either the maxilla, mandible, or both jaws, had been using complete dentures, and were seeking new complete dentures. The exclusion criteria were as follows: (1) individuals residing in or attending a nursing home; (2) individuals receiving dietary guidance from healthcare professionals; (3) individuals wearing implant overdentures; and (4) individuals unable to cooperate with dietary records owing to health conditions, such as dementia.

This study was conducted in accordance with the principles of the Declaration of Helsinki, approved by the Institutional Ethical Review Board of Showa University (2023-080-A), and registered with the Japan Registry of Clinical Trials (jRCT1032230495). Written informed consent was obtained from all participants before enrollment. This manuscript was reported in accordance with the Consolidated Standards of Reporting Trials (CONSORT) 2010 statement.

Sample size calculation

Based on a previous study [13], which reported an assumed effect size of 0.94 for differences in total fruit and vegetable intake between groups, a sample size was calculated using G*power software (Heinrich-Heine-Universität Düsseldorf, Germany). For the primary outcome of serum albumin levels, with the α set at 0.05 and the β set at 0.20, a minimum of 20 participants per group was determined. Anticipating a dropout rate of 20%, the required sample size was adjusted to 48 patients.

Randomization and intervention

**Figure 1:**
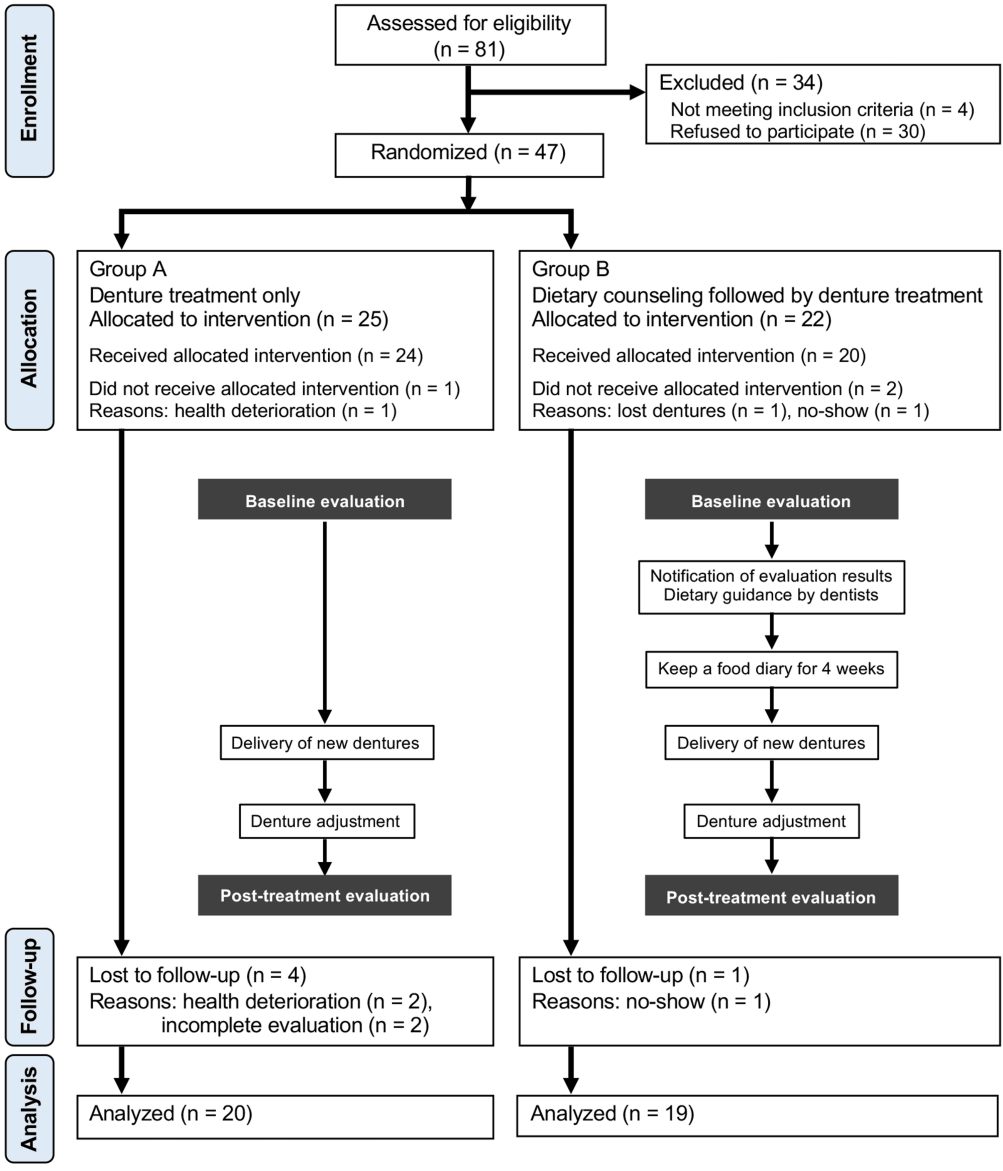
CONSORT 2010 flow diagram and study protocol CONSORT: Consolidated Standards of Reporting Trials

The participants were stratified by age and sex and randomly allocated to two groups using the permuted block method with concealment via software (Mujinwari; Iruka System Corporation, Tokyo, Japan) at a ratio of 1:1: Group A received denture treatment only, and Group B received dietary guidance followed by denture treatment (Figure 1). A single dentist (H.S.) handled the participant enrollment and software-based assignments. Owing to the nature of the dietary guidance provided, the participants were promptly informed of their group assignments, making blinding both participants and dentists unfeasible.

Initially, adjustments were made when the participants reported any pain from their current dentures, and treatment was provided for any remaining teeth as needed. These procedures were performed by five dentists, each with over 10 years of experience. Subsequently, a questionnaire was used to gather information on the participants' general health, living environment, and attitudes toward diet, followed by an oral examination. Baseline assessments included blood tests, dietary intake surveys, and masticatory function assessments.

Following these baseline assessments, dietary guidance was provided exclusively to Group B. For individualized dietary guidance, two trained dentists (H.S. and R.K.) served as advisers, reviewing the blood test results and BDHQ findings in advance and preparing a personalized, multi-page pamphlet based on the Japan Food Guide [22]. This pamphlet was tailored to address the patient's specific nutritional excesses and deficiencies. During the 15-minute session of individualized, simple dietary guidance, one of the advisers presented the blood test and BDHQ results, highlighted the identified imbalances, and used the pamphlet to provide a detailed explanation of the recommended dietary modifications. To monitor compliance, the participants maintained a food diary for four weeks after receiving the dietary guidance, submitting it at two and four weeks. The food diary contained a checklist of 10 food categories to record daily intake.

Following the baseline evaluations, complete dentures were fabricated for both groups using conventional methods by the same five dentists who performed denture adjustments and treated any remaining teeth. For patients requiring partial dentures on the opposite dental arch, partial dentures were fabricated simultaneously upon request. In Group B, dentures were delivered after the submission of the food diary. Adjustments were made to relieve any pain or discomfort, and once improvement was achieved, post-treatment evaluations were conducted using the same assessment measures as those at baseline. Consequently, the post-treatment evaluation was performed approximately two to three months after the denture replacement.

Questionnaires and oral examination

To investigate patient characteristics, an oral examination, interview, and self-administered questionnaires were administered to assess behavior and awareness of diet and nutrition. An oral examination was performed to evaluate the position of the edentulous jaw and the number of remaining teeth. Body mass index (BMI) was calculated based on height and weight. Medical history was reviewed, and the Charlson Comorbidity Index was used to score comorbidities [23]. The presence of polypharmacy was defined as the prescription of five or more medications. The participants were also asked about their household size and number of daily meals. They responded to the following questions: "To what extent do you pay attention to maintaining a healthy diet?" (attention to a healthy diet) and "How often do you check TV programs, magazines, the internet, or other sources related to health and nutrition?" (nutrition-related media consumption). Responses were collected using a four-point Likert scale and classified into high and low categories.

Blood biomarkers

The participants fasted for eight hours before blood sampling, during which only water was permitted. Blood samples were collected by clinical laboratory technicians in the morning and analyzed by BML, Inc. (Tokyo, Japan), who were blinded to the group allocation. Blood biomarkers that may reflect the nutritional status and a marker of inflammation were analyzed [19-21], including serum albumin, total protein, prealbumin (transthyretin), total cholesterol, total peripheral lymphocyte count, hemoglobin, CRP, and eight EAAs (threonine, valine, methionine, isoleucine, leucine, phenylalanine, tryptophan, and lysine). Additionally, the total EAAs (the sum of eight EAAs excluding histidine) and total branched-chain amino acids (BCAAs), including valine, isoleucine, and leucine, were calculated.

Dietary intake survey

The BDHQ was used to evaluate food and nutrient intake over the past month [24]. Based on previous reports [7,14], the daily intake of fat, saturated fat, protein, dietary fiber, fish with bones, non-oily fish, chicken, carrots/pumpkins, cabbage, and citrus fruits was calculated using a dedicated software that applied the residual method (Gender Medical Research, Inc., Tokyo, Japan).

Masticatory function

The masticatory function was objectively evaluated using the gummy jelly method and occlusal force and contact area measurements. The participants were instructed to freely chew a standardized gummy jelly (Glucolumn; GC Corporation, Tokyo, Japan) for 20 seconds, rinse their mouth with 10 mL of water, and immediately spit the mixture into a filtered cup. The glucose concentration of the filtrate was measured using a Glucosensor GS-II (GC Corporation). The occlusal force and contact area were calculated using dedicated software (GC Corporation) by having the participants clench a pressure-sensitive film (Dental Prescale II; GC Corporation) in the intercuspal position at the maximum force for three seconds. Measurements were performed twice with a three-minute interval, and the mean value was used [25].

Statistical analyses

Demographic data were compared between groups using the Mann-Whitney *U* test for continuous variables and the chi-square test for categorical variables. To assess the effects of each intervention, the baseline and post-treatment results within each group were compared using the Wilcoxon signed-rank test. To evaluate the impact of dietary guidance and denture treatment on blood biomarkers and dietary intake, between-group comparisons of changes from baseline to post-treatment were conducted using the Mann-Whitney *U* test. Due to the exploratory nature of this study, adjustments for multiple comparisons were not made. Statistical analyses were performed using JMP Pro (version 17.0; JMP Statistical Discovery LLC, Cary, NC, USA) with the significance level set at 5%.

## Results

Of the 81 eligible patients, 47 consented to participate in the study; however, eight participants withdrew (Figure 1). As a result, data from 39 participants, comprising 20 from Group A and 19 from Group B, were analyzed (17 males and 22 females; mean age 73.4 ± 10.6 years).

The patient characteristics are shown in Table 1. Of the 39 participants, 15 (38.5%) were completely edentulous. No significant differences were found in the patient characteristics.

**Table 1:** Patient characteristics ^a^ Data are presented as medians (first to third quartile). The Mann–Whitney *U* test was applied. ^b^ Data are presented as the number of participants (percentage). The chi-square test was applied. Group A received denture treatment only, and Group B received dietary guidance followed by denture treatment. BMI, body mass index.

Patient Characteristics	Total (n = 39)	Group A (n = 20)	Group B (n = 19)	P-value
Age (years) ^a^	75.0 (69.0 to 80.0)	74.5 (69.3 to 78.5)	77.0 (69.0 to 84.0)	0.221
Sex ^b^				
Male	17 (44%)	9 (45%)	8 (42%)	0.855
Female	22 (56%)	11 (55%)	11 (58%)	
Edentulous jaw ^b^				
Maxilla	20 (51%)	11 (55%)	9 (47%)	0.537
Mandible	4 (10%)	1 (5%)	3 (16%)	
Maxilla and mandible	15 (38%)	8 (40%)	7 (37%)	
BMI (kg/m^2^) ^a^	22.3 (19.6 to 24.4)	22.7 (19.8 to 24.8)	20.9 (19.2 to 23.6)	0.440
Charlson Comorbidity Index ^a^	0 (0 to 1)	0 (0 to 1)	0 (0 to 0)	0.129
Polypharmacy ^b^				
≥ 5	13 (33%)	6 (30%)	7 (37%)	0.651
< 4	26 (67%)	14 (70%)	12 (63%)	
Household size ^a^	2 (1 to 3)	2 (1 to 3)	2 (2 to 3)	0.365
Number of meals per day ^b^				
1 meal	1 (3%)	1 (5%)	0 (0%)	0.468
2 meals	10 (26%)	6 (30%)	4 (21%)	
3 meals	28 (72%)	13 (65%)	15 (79%)	
Attention to a healthy diet ^b^				
High	9 (23%)	5 (25%)	4 (21%)	0.770
Low	30 (77%)	15 (75%)	15 (79%)	
Nutrition-related media consumption ^b^				
High	29 (74%)	14 (70%)	15 (79%)	0.522
Low	10 (26%)	6 (30%)	4 (21%)	
Number of remaining teeth ^a^	4 (0 to 10)	4 (0 to 9)	5 (0 to 11)	0.361

In Group A, the blood biomarkers, nutrient intake, and dietary intake showed no significant improvements between baseline and post-treatment, although the valine level decreased after treatment (Table 2). In Group B, no significant differences were found in the blood biomarkers and nutrient intake between baseline and post-treatment; however, the dietary intake of fish with bones and non-oily fish significantly increased after treatment (Table 3). Regarding the objective masticatory function, the occlusal force significantly increased in both groups after the intervention, and the occlusal contact area significantly increased in Group B.

**Table 2:** Comparison of baseline and post-treatment results in Group A * P < 0.05 Group A received denture treatment only. Data are presented as medians (first to third quartile). The baseline and post-treatment results were compared using the Wilcoxon signed-rank test. The effect size was calculated via r = z/√n (r, effect size; z, z-score; n, observation number). EAA, essential amino acid; BCAA, branched-chain amino acid.

Parameters (n = 20)	Baseline	Post-treatment	Effect Size	P-value
Blood biomarkers				
Albumin (g/dL)	4.3 (4.0 to 4.5)	4.3 (4.1 to 4.5)	0.154	0.523
Total protein (g/dL)	7.0 (6.8 to 7.3)	7.1 (6.8 to 7.2)	0.208	0.365
Prealbumin (mg/dL)	26.8 (24.3 to 33.0)	26.3 (23.5 to 33.9)	0.004	0.993
Total cholesterol (mg/dL)	199.5 (175.8 to 220.3)	185.0 (172.8 to 210.0)	0.267	0.242
Total peripheral blood lymphocyte count (/μL)	1644.0 (1246.8 to 2569.5)	1515.5 (1098.5 to 2455.0)	0.351	0.123
Hemoglobin (g/dL)	14.3 (12.7 to 14.8)	14.0 (12.2 to 14.8)	0.235	0.311
C-reactive protein (mg/dL)	0.07 (0.04 to 0.11)	0.06 (0.03 to 0.12)	0.348	0.126
Threonine (nmol/mL)	140.9 (123.2 to 171.0)	127.1 (109.1 to 175.6)	0.351	0.123
Valine (nmol/mL)	249.5 (217.3 to 295.5)	243.7 (220.1 to 258.8)	0.267	0.246
Methionine (nmol/mL)	27.1 (24.6 to 29.2)	24.0 (20.7 to 27.0)	0.555	0.011*
Isoleucine (nmol/mL)	71.7 (56.2 to 84.7)	64.7 (56.1 to 75.4)	0.401	0.076
Leucine (nmol/mL)	125.9 (102.9 to 147.3)	121.1 (108.3 to 133.4)	0.192	0.409
Phenylalanine (nmol/mL)	63.2 (56.6 to 70.0)	60.2 (54.4 to 64.6)	0.371	0.100
Tryptophan (nmol/mL)	42.5 (38.6 to 49.7)	41.4 (33.6 to 44.1)	0.288	0.206
Lysine (nmol/mL)	210.5 (187.0 to 231.3)	199.0 (169.6 to 219.4)	0.384	0.090
EAAs (nmol/mL)	918.7 (846.7 to 1039.2)	864.2 (799.4 to 963.0)	0.392	0.083
BCAAs (nmol/mL)	451.8 (375.3 to 527.7)	425.8 (374.6 to 466.4)	0.259	0.261
Daily nutrient intake				
Fat (g/day)	58.8 (49.4 to 67.6)	60.0 (53.9 to 73.4)	0.305	0.180
Saturated fat (g/day)	15.3 (12.9 to 18.6)	15.7 (12.9 to 20.5)	0.313	0.168
Protein (g/day)	79.3 (71.1 to 89.4)	75.4 (68.1 to 100.7)	0.063	0.791
Total dietary fiber (g/day)	11.3 (9.8 to 14.6)	13.4 (10.2 to 15.9)	0.322	0.156
Daily dietary intake				
Fish with bones (g/day)	5.9 (0.0 to 36.6)	6.6 (1.0 to 20.1)	0.162	0.495
Non-oily fish (g/day)	17.2 (9.3 to 25.1)	18.9 (7.7 to 32.9)	0.162	0.490
Chicken (g/day)	23.2 (17.2 to 39.9)	26.6 (7.6 to 48.3)	0.018	0.953
Carrots and pumpkins (g/day)	12.4 (9.3 to 19.2)	12.7 (6.8 to 25.8)	0.229	0.325
Cabbage (g/day)	23.3 (14.3 to 51.6)	34.1 (9.3 to 56.2)	0.192	0.409
Citrus fruits (g/day)	2.6 (0.0 to 23.6)	2.2 (0.0 to 26.1)	0.021	0.952
Objective masticatory function				
Masticatory performance (mg/dL)	109.5 (75.8 to 143.8)	136.5 (93.3 to 151.0)	0.347	0.125
Occlusal force (N)	159.0 (96.8 to 252.9)	222.6 (169.4 to 340.6)	0.584	0.007*
Occlusal contact area (mm^2^)	4.9 (2.9 to 7.5)	5.6 (4.0 to 8.5)	0.359	0.112

**Table 3:** Comparison of the baseline and post-treatment results in Group B * P < 0.05 Group B received dietary guidance followed by denture treatment. Data are presented as medians (first to third quartile). The baseline and post-treatment results were compared using the Wilcoxon signed-rank test. The effect size was calculated via r = z/√n (r, effect size; z, z-score; n, observation number). EAA, essential amino acid; BCAA, branched-chain amino acid.

Parameters (n = 19)	Baseline	Post-treatment	Effect Size	P-value
Blood biomarkers				
Albumin (g/dL)	4.2 (3.9 to 4.3)	4.0 (3.9 to 4.4)	0.113	0.642
Total protein (g/dL)	7.3 (6.7 to 7.5)	7.0 (6.7 to 7.3)	0.257	0.267
Prealbumin (mg/dL)	26.5 (22.1 to 29.9)	27.6 (21.1 to 29.7)	0.065	0.798
Total cholesterol (mg/dL)	212.0 (174.0 to 234.0)	200.0 (169.0 to 244.0)	0.148	0.534
Total peripheral blood lymphocyte count (/μL)	1500.0 (1360.0 to 1751.0)	1451.0 (1140.0 to 761.0)	0.185	0.441
Hemoglobin (g/dL)	13.3 (12.4 to 14.2)	12.8 (12.1 to 14.3)	0.196	0.409
C-reactive protein (mg/dL)	0.08 (0.05 to 0.14)	0.07 (0.05 to 0.11)	0.256	0.279
Threonine (nmol/mL)	120.7 (105.3 to 140.3)	125.9 (99.9 to 136.4)	0.037	0.891
Valine (nmol/mL)	240.6 (203.5 to 263.5)	234.8 (208.0 to 270.6)	0.074	0.768
Methionine (nmol/mL)	25.6 (19.7 to 27.2)	25.7 (21.4 to 30.2)	0.282	0.229
Isoleucine (nmol/mL)	62.4 (54.3 to 68.8)	62.3 (54.6 to 68.4)	0.148	0.541
Leucine (nmol/mL)	121.9 (103.5 to 139.9)	122.0 (105.4 to 144.9)	0.212	0.358
Phenylalanine (nmol/mL)	66.1 (58.6 to 70.1)	68.4 (56.4 to 72.0)	0.134	0.574
Tryptophan (nmol/mL)	39.5 (33.9 to 46.6)	41.2 (33.7 to 44.7)	0.083	0.731
Lysine (nmol/mL)	206.5 (188.0 to 224.8)	209.8 (194.0 to 230.4)	0.275	0.251
EAAs (nmol/mL)	859.2 (826.8 to 945.8)	889.9 (809.1 to 971.6)	0.277	0.241
BCAAs (nmol/mL)	435.0 (367.3 to 478.0)	398.6 (374.1 to 483.8)	0.111	0.651
Daily nutrient intake				
Fat (g/day)	66.1 (58.6 to 76.3)	63.5 (53.4 to 76.1)	0.106	0.658
Saturated fat (g/day)	19.7 (16.2 to 22.4)	17.1 (14.5 to 21.9)	0.217	0.358
Protein (g/day)	82.1 (70.5 to 88.2)	85.2 (71.9 to 101.0)	0.185	0.441
Total dietary fiber (g/day)	13.4 (9.6 to 15.4)	13.6 (11.0 to 15.9)	0.295	0.206
Daily dietary intake				
Fish with bones (g/day)	0.0 (0.0 to 12.9)	6.6 (0.0 to 30.8)	0.482	0.035*
Non-oily fish (g/day)	14.1 (6.3 to 22.5)	20.5 (10.1 to 47.3)	0.495	0.030*
Chicken (g/day)	32.8 (13.6 to 43.8)	27.4 (12.3 to 39.6)	0.215	0.369
Carrots and pumpkins (g/day)	18.1 (10.2 to 32.7)	23.6 (15.0 to 29.7)	0.028	0.922
Cabbage (g/day)	27.8 (5.7 to 60.1)	41.4 (28.7 to 76.3)	0.293	0.211
Citrus fruits (g/day)	5.8 (0.0 to 18.8)	5.9 (0.0 to 51.2)	0.425	0.068
Objective masticatory function				
Masticatory performance (mg/dL)	132.0 (51.0 to 161.0)	133.0 (90.0 to 169.0)	0.231	0.332
Occlusal force (N)	133.8 (71.1 to 269.4)	249.7 (150.2 to 333.6)	0.572	0.011*
Occlusal contact area (mm^2^)	4.6 (2.0 to 7.8)	7.0 (4.9 to 9.5)	0.517	0.023*

The between-group comparisons of changes from baseline to post-treatment are shown in Table 4. Group B exhibited significantly greater increases in methionine, lysine, and total EAAs and in the intake of fish with bones.

**Table 4:** Comparison of changes in blood biomarkers, nutrient intake, and dietary intake between groups * P < 0.05 Group A received denture treatment only, and Group B received dietary guidance followed by denture treatment. Data are presented as medians (first to third quartile). Between-group comparisons of changes from baseline to post-treatment were conducted using the Mann–Whitney *U* test. The effect size was calculated via r = z/√n (r, effect size; z, z-score; n, observation number). EAA, essential amino acid; BCAA, branched-chain amino acid.

Parameters	Group A (n=20)	Group B (n=19)	Effect Size	P-value
Blood biomarkers				
Albumin (g/dL)	0.0 (−0.1 to 0.3)	0.0 (−0.2 to 0.2)	0.125	0.444
Total protein (g/dL)	−0.1 (−0.4 to 0.3)	0.1 (−0.3 to 0.3)	0.000	1.000
Prealbumin (mg/dL)	0.0 (−2.5 to 1.7)	−0.3 (−4.6 to 2.1)	0.029	0.866
Total cholesterol (mg/dL)	−1.5 (−24.0 to 4.0)	−5.0 (−20.0 to 17.0)	0.065	0.694
Total peripheral blood lymphocyte count (/μL)	−135.5 (−308.8 to 102.0)	−27.0 (−300.0 to 174.0)	0.045	0.790
Hemoglobin (g/dL)	−0.1 (−0.7 to 0.3)	−0.2 (−0.8 to 0.5)	0.018	0.922
C-reactive protein (mg/dL)	−0.01 (−0.03 to 0.20)	0.00 (−0.06 to 0.02)	0.011	0.955
Threonine (nmol/mL)	−16.4 (−39.4 to 8.1)	−2.9 (−13.1 to 16.9)	0.248	0.126
Valine (nmol/mL)	−11.8 (−38.1 to 12.7)	0.3 (−21.7 to 29.2)	0.184	0.255
Methionine (nmol/mL)	−4.5 (−8.6 to −0.3)	1.5 (−1.2 to 3.1)	0.405	0.012*
Isoleucine (nmol/mL)	−4.3 (−20.9 to 2.5)	2.3 (−7.3 to 12.2)	0.238	0.140
Leucine (nmol/mL)	−4.9 (−19.4 to 17.5)	1.8 (−4.7 to 20.6)	0.220	0.173
Phenylalanine (nmol/mL)	−3.8 (−12.4 to 3.2)	0.3 (−2.2 to 6.2)	0.315	0.051
Tryptophan (nmol/mL)	−1.8 (−5.3 to 1.7)	0.3 (−3.4 to 3.4)	0.209	0.196
Lysine (nmol/mL)	−9.4 (−38.5 to 6.9)	4.3 (−4.3 to 22.7)	0.319	0.048*
EAAs (nmol/mL)	−65.0 (−159.6 to 32.5)	20.7 (−15.3 to 72.3)	0.351	0.029*
BCAAs (nmol/mL)	−25.2 (−78.6 to 34.6)	5.8 (−32.8 to 55.4)	0.214	0.187
Daily nutrient intake				
Fat (g/day)	2.1 (−3.8 to 11.4)	−0.7 (−10.1 to 6.6)	0.218	0.177
Saturated fat (g/day)	0.7 (−0.2 to 2.9)	−1.1 (−3.1 to 1.7)	0.245	0.129
Protein (g/day)	−2.1 (−9.1 to 16.2)	1.9 (−13.4 to 12.3)	0.065	0.694
Total dietary fiber (g/day)	2.1 (−1.5 to 4.0)	1.8 (−1.8 to 3.0)	0.137	0.399
Daily dietary intake				
Fish with bones (g/day)	0.0 (−13.2 to 5.9)	4.6 (0.0 to 16.2)	0.326	0.043*
Non-oily fish (g/day)	1.6 (−2.4 to 7.3)	11.4 (−6.4 to 22.9)	0.234	0.148
Chicken (g/day)	−0.1 (−17.9 to 15.7)	−2.4 (−21.3 to 12.7)	0.103	0.527
Carrots and pumpkins (g/day)	1.0 (−3.1 to 11.4)	−0.5 (−7.5 to 10.1)	0.074	0.653
Cabbage (g/day)	6.0 (−14.6 to 28.2)	5.8 (−5.3 to 17.7)	0.032	0.855
Citrus fruits (g/day)	0.0 (−5.4 to 14.4)	0.2 (0.0 to 25.2)	0.182	0.262

## Discussion

This study revealed a significant increase in occlusal force after denture treatment in both groups; however, no significant improvements were observed in the blood biomarkers or nutrient intake. Furthermore, the results showed that providing dietary guidance led to greater increases in the EAA levels of methionine and lysine and in the consumption of fish with bones compared to prosthetic treatment alone. A single-arm prospective study that combined dietary counseling with partial denture treatment found similar results, reporting no hematological changes in carotenoid and vitamin C levels but an improvement in vegetable intake [17]. These findings suggest that denture treatment significantly improves masticatory function and that combining dietary guidance with prosthetic treatment may help improve dietary habits and enhance nutritional status, although dietary guidance appears to have a limited impact on blood biomarkers. Further, these results suggest that dietary guidance may encourage a greater inclination toward including fish in meals, thereby enhancing dietary diversity in denture users.

As mentioned above, the group that received dietary guidance showed significantly greater improvements in the EAA levels of methionine and lysine, as well as an increase in the intake of fish with bones. Methionine is commonly found in fish, meat, and certain vegetables, whereas lysine is more abundant in meat, fish, and dairy products [26]. Since EAAs cannot be synthesized by the body and must be obtained through diet, deficiencies can disrupt the amino acid balance and negatively impact nutritional status. Regarding the effect of dietary guidance combined with prosthetic treatment, previous studies on partial denture wearers found that dietary guidance improved vegetable intake [17] and nutritional status, as assessed by the MNA [16], although blood biomarkers remained constant [17]. Further, studies on complete denture wearers have reported improved intake of fruits, vegetables [13], chicken, and fish [14], although none of the studies measured blood parameters. The findings of the present study also suggest that dietary guidance may help increase food intake and dietary diversity, potentially leading to increased EAA levels. The dietary guidance in the present study is unique because it can be implemented by dentists or dental hygienists based on the assessment of the patient's nutritional status and eating behavior.

The present study differs from previous research, where dentists provided two dietary advice sessions after prosthetic treatment using a pamphlet [17] or one session immediately after treatment using a uniform pamphlet [14], by offering dietary guidance prior to the prosthetic treatment and following up with a four-week food diary record. For better improvement of diet or nutrition, the timing of dietary guidance and the method and duration of follow-up should be further examined. As regular nutritional monitoring and individualized nutritional therapy are critical for managing nutrition in older adults [27], it is essential to assess the nutritional status of dental patients along with their oral frailty. A continuous approach is necessary to improve the nutritional status of malnourished patients.

This study has certain limitations. First, as an exploratory rather than a confirmatory study, the findings should be interpreted with caution, and the generalizability is limited. Therefore, the potential improvement in the EAA levels requires further investigation. Second, for feasibility purposes, participants with at least one edentulous jaw were eligible for inclusion, and only 38.5% wore complete dentures for both the upper and lower jaws. This variation in the oral condition may have influenced the outcomes related to masticatory function. Third, no placebo was established, as only Group B received dietary guidance, whereas Group A received no intervention. Therefore, further studies under controlled conditions are warranted. Fourth, post-treatment evaluations were conducted two to three months after denture delivery, resulting in a relatively short follow-up period. Longer-term studies are required to assess these sustained effects. Lastly, this study was designed to examine the effect of dietary guidance that can be implemented in dental clinics rather than that of strictly supervised dietary management. Therefore, caution must be exercised in generalizing the results of this study.

## Conclusions

This is the first exploratory study to examine the changes in nutrition-related blood biomarkers among edentulous patients receiving both dietary guidance and denture treatment. Following prosthetic treatment, the occlusal force significantly increased; however, blood biomarkers did not improve, even when dietary guidance was provided. Meanwhile, fish intake significantly increased in patients who received dietary guidance. Additionally, dietary guidance before denture treatment led to significantly greater changes in EAA levels, specifically in methionine and lysine levels, as measured by blood tests. These findings highlight the critical role of dietary guidance in enhancing dietary habits and supporting the nutritional status of dental patients seeking prosthetic rehabilitation.
